# [Corrigendum] PRIMA‑1^met^ induces autophagy in colorectal cancer cells through upregulation of the mTOR/AMPK‑ULK1‑Vps34 signaling cascade

**DOI:** 10.3892/or.2024.8703

**Published:** 2024-01-17

**Authors:** Xiao-Lan Li, Jianbiao Zhou, Chen-Jing Xia, Han Min, Zhong-Kai Lu, Zhi-Rong Chen

Oncol Rep 45: 86, 2021; DOI: 10.3892/or.2021.8037

Following the publication of the above article, the authors contacted the Editorial Office to explain that the strips of β-actin, LC3 and p62 proteins of the RKO cell line shown in Fig. 2A and B, and those of the SW620 cell line shown in Fig. 3A and B, were assembled in these figures incorrectly. To rectify the presentation of these two figures, the authors proposed that they replace the strips of β-actin and p62 proteins in the original Figs. 2B and 3B with the β-actin bands from one of the repeated western blotting experiments.

The revised and corrected versions of Figs. 2 and 3 are shown on the next page. The authors wish to emphasize that these corrections do not grossly affect either the results or the conclusions reported in this work. The authors all agree to the publication of this Corrigendum, and are grateful to the Editor of *Oncology Reports* for granting them the opportunity to correct the errors that were made during the assembly of these figures. Lastly, the authors apologize to the readership for any inconvenience these errors may have caused.

## Figures and Tables

**Figure 2.**
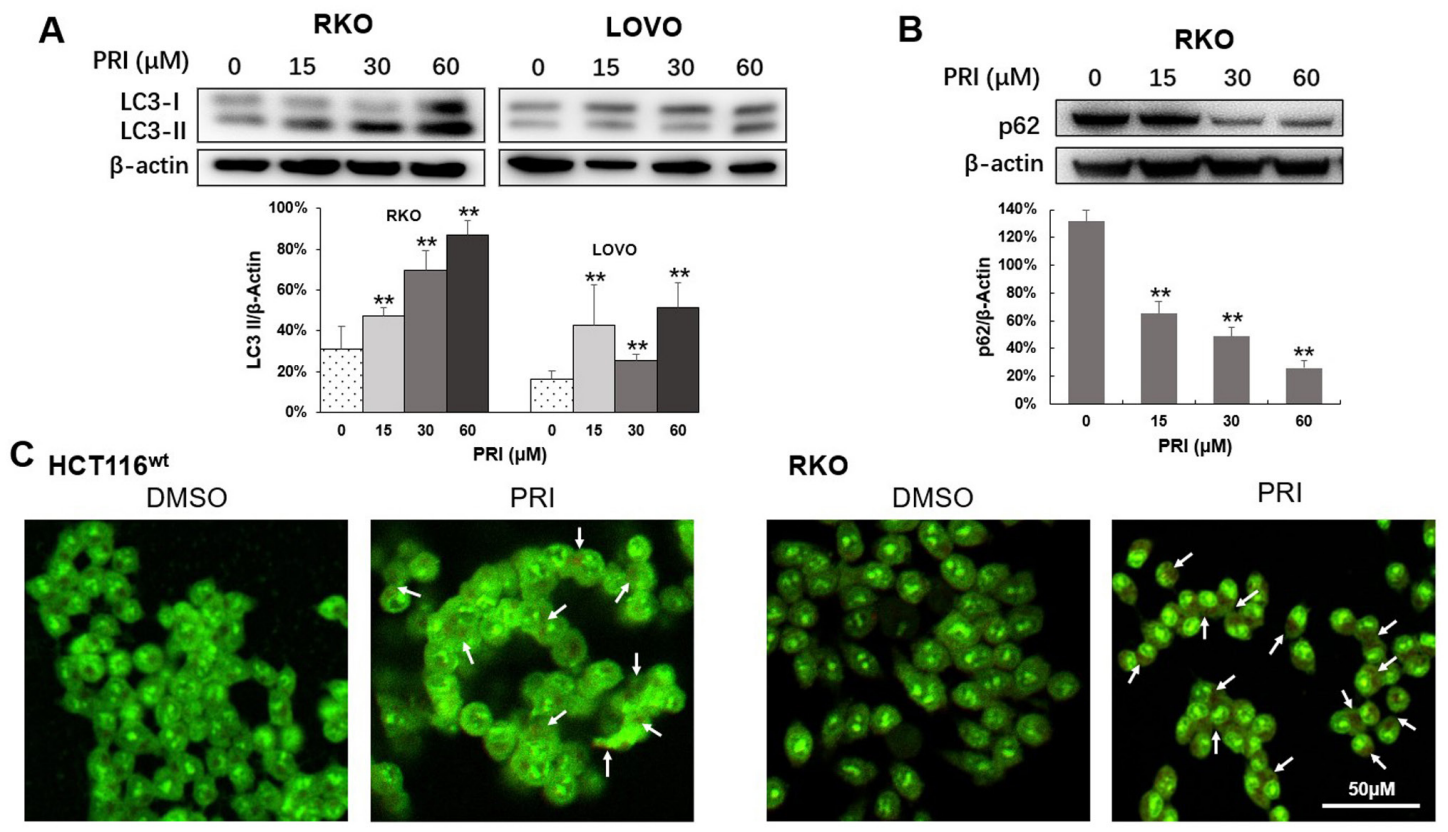
PRI promotes autophagy flux in CRC cell lines with wild-type p53. (A) LC3-I and LC3-II were assessed via western blotting in RKO and LOVO cell lysates following 24 h treatment with PRI. The quantity of LC3-II was calculated after three independent experiments. The amount of LC3-II was normalized to β-actin and data are presented as the mean + SEM. **P<0.01, compared with the PRI untreated cells. (B) The expression of p62 was decreased in RKO cells following PRI treatment in a dose-dependent manner. Data are presented as the mean + SEM (n=3). **P<0.01, compared with the untreated group. (C) HCT116wt and RKO cells were treated with 30 µM PRIMA-1met, after which fluorescence was evaluated. The results revealed greater red fluorescence in acidic vesicular organelles stained with acridine orange (white arrow). The fluorescence intensities were also estimated and presented as the mean + SEM (n=3). **P<0.01, compared with the DMSO-treated group. PRI, p53-reactivation and induction of massive apoptosis-1, APR-017 methylated; CRC, colorectal cancer.

**Figure 3.**
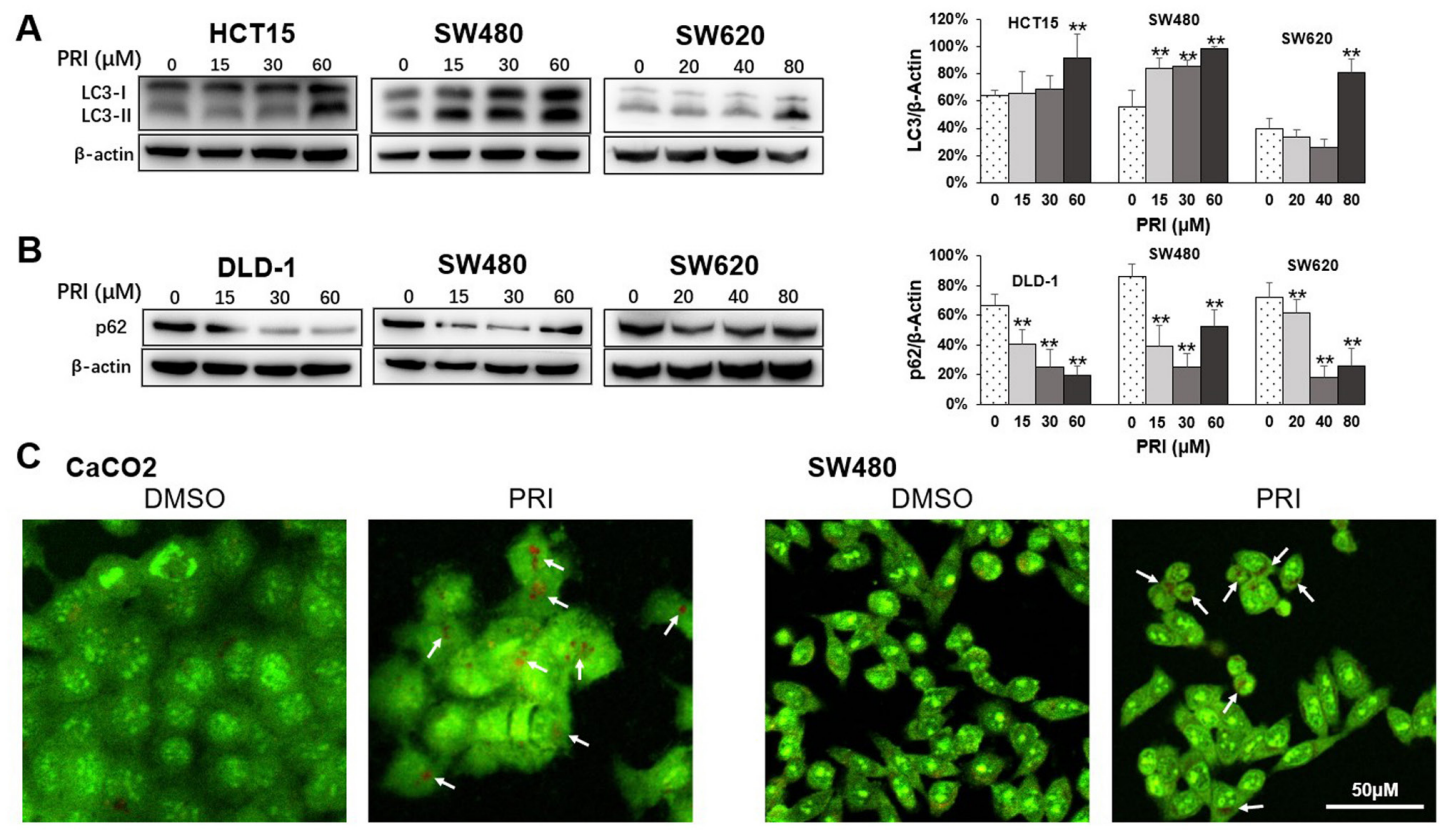
PRI promotes autophagy flux in CRC cell lines with mutant p53. (A) Western blotting was used to assess LC3-I and LC3-II expression in HCT15, SW480 and SW620 cell lysates treated with different concentrations of PRI for 24 h. Gray values represent the quantity of LC3-II normalized to that of respective β-actin and assessed as the mean + SEM (n=3). **P<0.01, compared with the PRI untreated group. (B) p62 levels were decreased in DLD-1, SW480 and SW620 cell treated with PRI for 24 h. Data are presented as the mean + SEM (n=3). *P<0.05, **P<0.01, compared with the untreated group. (C) Greater red fluorescence in acidic vesicular organelles was observed in CaCO2 and SW480 cells treated with 30 µM PRI following acridine orange staining (white arrow). The fluorescence intensities were obtained and presented as the mean + SEM (n=3). **P<0.01, compared with the DMSO-treated group. PRI, p53-reactivation and induction of massive apoptosis-1, APR-017 methylated; CRC, colorectal cancer.

